# Compressive Cervical Myelopathy in Patients With Demyelinating Disease of the Central Nervous System: Improvement After Surgery Despite a Late Diagnosis

**DOI:** 10.7759/cureus.13161

**Published:** 2021-02-05

**Authors:** Carl Youssef, Umaru Barrie, Mahmoud Elguindy, Zachary Christian, James P Caruso, Zachary D Johnson, Kristen Hall, Salah G Aoun, Carlos A Bagley, Mazin Al Tamimi

**Affiliations:** 1 Neurological Surgery, University of Texas Southwestern Medical Center, Dallas, USA

**Keywords:** multiple sclerosis, transverse myelitis, demyelinating disorders, cervical spondylosis, anterior cervical discectomy, cervical spine fusion

## Abstract

Objective

We aimed to assess the impact of surgical intervention on outcome in patients diagnosed with demyelinating disorders and cervical degenerative disease warranting surgical intervention.

Methods

The records of patients with a diagnosis of a demyelinating disorder of the central nervous system who underwent cervical spine surgery at a single institution from 2016 to 2020 were reviewed. Demyelinating disease included multiple sclerosis (MS), neuromyelitis optica, and transverse myelitis (TM). The dates of initial spine symptom onset, recognition of spinal pathology by the primary provider, referral to spine surgery, and spine surgery procedures were collected. Hospital length of stay (LOS) and postoperative outcomes and complications were recorded.

Results

A total of 19 patients with a diagnosis of demyelinating disorders underwent cervical spine surgery at our institution. Seventeen patients had MS. The average time interval between a documented diagnosis of myelopathy or radiculopathy and referral to the Spine clinic was 67.95 months (M=40, SD=64.87). Twelve patients had imaging studies depicting degenerative spine disease that would warrant surgical intervention at the time of examination by their primary physician. The average delay for referral to the Spine clinic for these patients was 16.5 months (M=5; SD=25.36). More than 89% of patients experienced significant neurologic improvement postoperatively.

Conclusions

There is a delay in the recognition of cervical spine disease amenable to a surgical resolution in patients with demyelinating disorders. Surgical treatment can lead to significant clinical improvement in this patient population even if delayed, and likely carries similar risk to that of the general population.

## Introduction

Demyelinating disorders consist of a wide spectrum of diseases resulting in the destruction of neuronal myelin sheath. Multiple sclerosis (MS) is the most common of these disorders and affects approximately 400,000 people in the United States [1]. The clinical presentation of MS is highly variable, but symptoms often include sensory changes, motor deficits, gait imbalance, and bowel or bladder dysfunction [2], many of which are also common in patients with cervical spondylotic myelopathy or radiculopathy. The coincidence of cervical spondylosis (CS) causing neural element compression and demyelinating disorders (DD) can blur the clinical picture and make surgical decision-making difficult. Prior studies have demonstrated that patients with DD and CS may benefit from surgical decompression [3-7]. However, it is still unclear how this combination delays the appropriate diagnosis and treatment of surgical spine disease, and whether surgical treatment can still clinically benefit patients despite this delay.

In this study, we analyzed the timeline of patients with coexistent CS and DD (DD/CS) spanning four crucial timestamps: 1- The initial appearance of neural compression-related symptoms, 2- The documented recognition of these symptoms by a medical specialist, 3- Patient referral to the Spine specialty clinic and 4- The date of surgery. We also assessed postoperative patient outcomes, including improvement in function, complications, and hospital length of stay. We hypothesized that there would be a significant delay in the recognition and treatment of cervical spondylosis in patients with DD, and that the delay in surgical treatment could mitigate the benefits of surgical decompression in this vulnerable patient population with preexisting underlying neurologic deficits.

## Materials and methods

Protocol and inclusion criteria

We conducted a retrospective review of patients with a diagnosis of DD who underwent elective cervical spine surgery for spondylosis between January 2016 and January 2020 at a single center institution. Demyelinating disorders included multiple sclerosis, transverse myelitis, and neuromyelitis optica, all of which were confirmed by our Neurology department. MS was diagnosed using the McDonald criteria [8]. Spinal procedures included both anterior and posterior cervical decompression and/or fusion. Patients who underwent surgeries for neoplasms, trauma, or infection were not included. Patient consent was not required for retrospective data pooling as patient data were deidentified once collected as is standard at our institution. The study protocol was approved by our institutional review board (STU-2020-0515).

Demographic and clinical data

Demographic data included age at the time of surgery, gender, and race. All imaging studies were reviewed independently by the authors to determine whether surgical pathology was present at the time of the patient’s initial presentation to the primary care physician. Surgical pathology was defined as severe central or neuroforaminal stenosis due to disc herniation, osteophyte formation, and/or ligamentous hypertrophy or ossification. Timeline details from initial symptom onset to surgical treatment were collected for each group, which included the following dates: the initial appearance of neural compression symptoms (myelopathy, radiculopathy or both), the initial visit with the physician office that diagnosed and documented these symptoms, the earliest availability of radiographic imaging showing cervical disease warranting surgery, the date of referral to the Spine clinic, and the date of surgery.

Postoperative outcomes

Neurologic status postoperatively was classified into three categories: “near-resolution” of preoperative symptoms, “partial improvement”, and “no change”. This data was based on the subjective self-assessment of the patient and on the neurological exam at the six-month clinic visit with the spine surgeon. All patients undergoing spine surgery at our institution are enrolled in our Enhanced Recovery After Surgery (ERAS) program which has been published elsewhere. Preoperative and postoperative patient assessment is done using the Patient-Reported Outcomes Measurement Information System 29 (PROMIS-29) as well as the Japanese Orthopedic Associate (JOA) myelopathy scale by physical therapy prior to surgery and during rehabilitation. We elected to simplify our classification system into three categories that were based on clinical exam in clinic in this study to provide clear categories of improvement. Near resolution involves an asymptomatic patient with full motor strength and minor residual paresthesia. No change signifies no motor improvement six months after surgery. Partial improvement signifies motor improvement from preoperative baseline after surgery. All patients in this series were followed for more than six months after surgery. Postoperative hospital length of stay was recorded. Patient records were also reviewed for postoperative wound infection, symptomatic pseudoarthrosis, or exacerbation of preexisting demyelinating symptoms.

Statistical analysis

Descriptive statistics included mean, median and standard deviation (SD). Our sample was too small to allow for analytical statistics between subgroups.

## Results

Demographic characteristics

A total of 19 patients with DD who underwent cervical spine surgery were identified to be included in the study (Table 1).

**Table 1:** Demographic, pre-operative clinical data, and post-operative outcomes of DD patients who underwent spine surgery

Total Number of Patients n (%)		19
Age (SD; range)		53.80 (13.63; 32 - 75)
Gender	Female	16 (84.21%)
	Male	3 (15.79%)
Race/Ethnicity	White	15 (78.95%)
	Black	4 (21.05%)
Pre-operative symptoms	Myelopathy	14 (68%)
	Myeloradiculopathy	2 (10.53%)
	Radiculopathy	3 (15.79%)
Spine disease present in initial imaging when seen by primary physician	Yes	12 (60%)
	No	8 (40%)
Post-operative Course	Near-resolution	8 (42.1%)
	Partial Improvement	9 (47.36%)
	No Change	2 (10.52%)

The cohort consisted of 16 females and three males, predominantly Caucasian and with an average age of 53.8 years. Seventeen patients had a diagnosis of multiple sclerosis, one had transverse myelitis, and one had neuromyelitis optica. Fourteen patients presented with myelopathy, two with myeloradiculopathy and three with radiculopathy. A total of 17 patients underwent an anterior approach to the spine, one patient underwent a posterior approach, and one a combined anterior and posterior fusion.

Patient outcomes

Overall, DD/CS patients experienced significant delays in care for symptoms related to CS. There was an average interval of 98.55 months between the appearance of spinal or nerve compression symptoms and the referral to the Neurosurgery Spine clinic (Table 2).

**Table 2:** Intervals between initial symptom appearance and surgical date

Interval	Time, in months, mean	Time, in months, median	SD	Range
Onset of spine-related symptoms to spine surgical referral	98.55	52	123.73	0 - 452
Documented diagnosis of symptoms to Spine Clinic referral	67.95	40	64.87	0 - 182
Imaging showing surgical spine disease to Spine Clinic referral	12.70	3	21.89	0 - 71
Spine clinic visit to date of surgery	10.45	2	17.17	1 - 56

Once the symptoms were documented by the primary treating physician, an average delay of 67.95 (M=40) months until referral to the Spine clinic was present. A delay of 12.7 (M=3) months was recorded for the entire cohort between the obtention of imaging showing neural compression warranting surgery and referral to a Spine specialist. Finally, an average time interval of 10.45 (M=2) months was present between referral to the Spine clinic and the date of surgery. A total of 12 patients already had imaging studies depicting degenerative spine disease that would warrant surgical intervention at the time of diagnosis of myelopathy or radiculopathy by their primary physician, and the average delay for referral to the Spine clinic for these patients was 16.5 months (M=5; SD=25.36).

Postoperative data

Seventeen patients reported neurologic improvement postoperatively, eight of whom had near-resolution of their preoperative symptoms. Two patients reported no improvement but had no worsening in their neurological function after surgery. One patient suffered from a transient C5 palsy after a multilevel posterior cervical decompression and fusion. His palsy resolved within three months after surgery. The average hospital length of stay was two days (M=2, Range 1-5). One patient had to be admitted to the ICU for airway observation after combined anterior and posterior approach and was discharged to home a day later. No patients had wound infections warranting readmission, or symptomatic pseudarthrosis requiring reoperation within six months after surgery.

Case illustration

**Figure 1:**
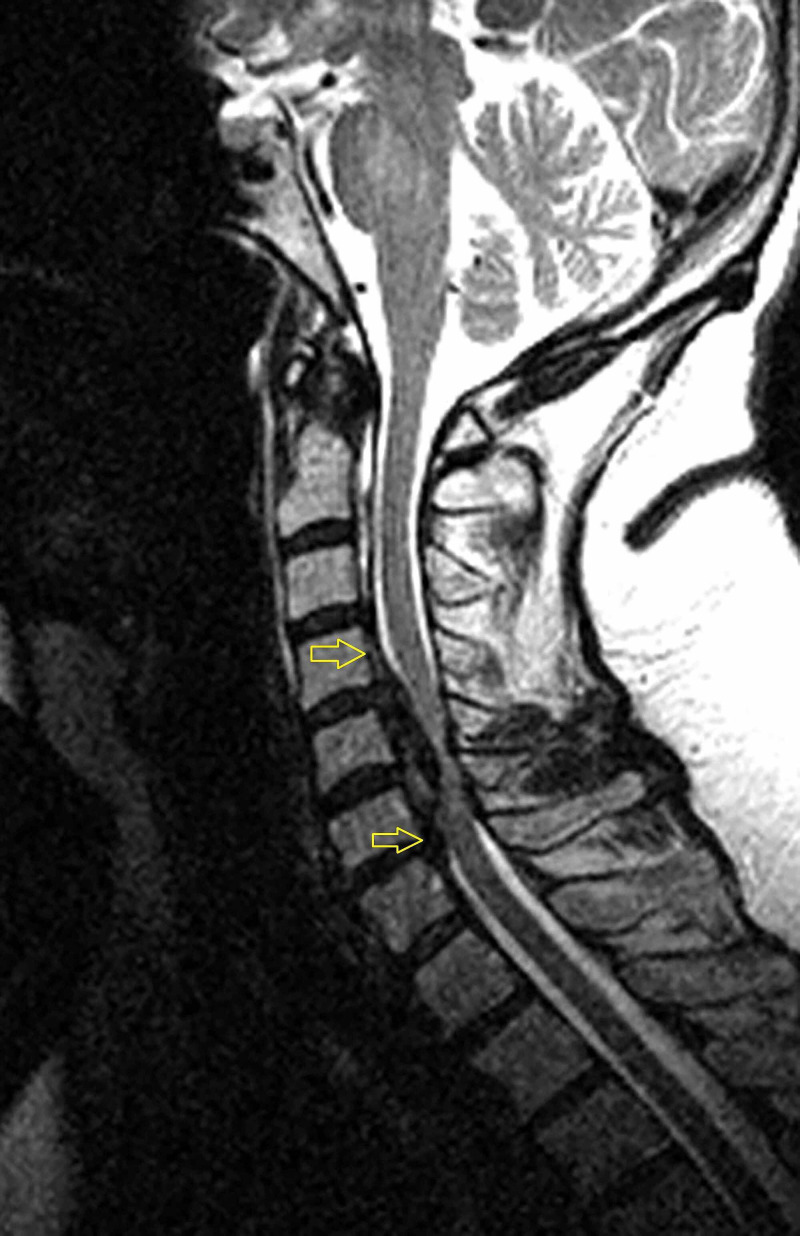
Sagittal T2 sequence magnetic resonance imaging scan showing severe spinal cord impingement by anterior compressive disease spanning C4 to C6 (yellow arrows).

**Figure 2:**
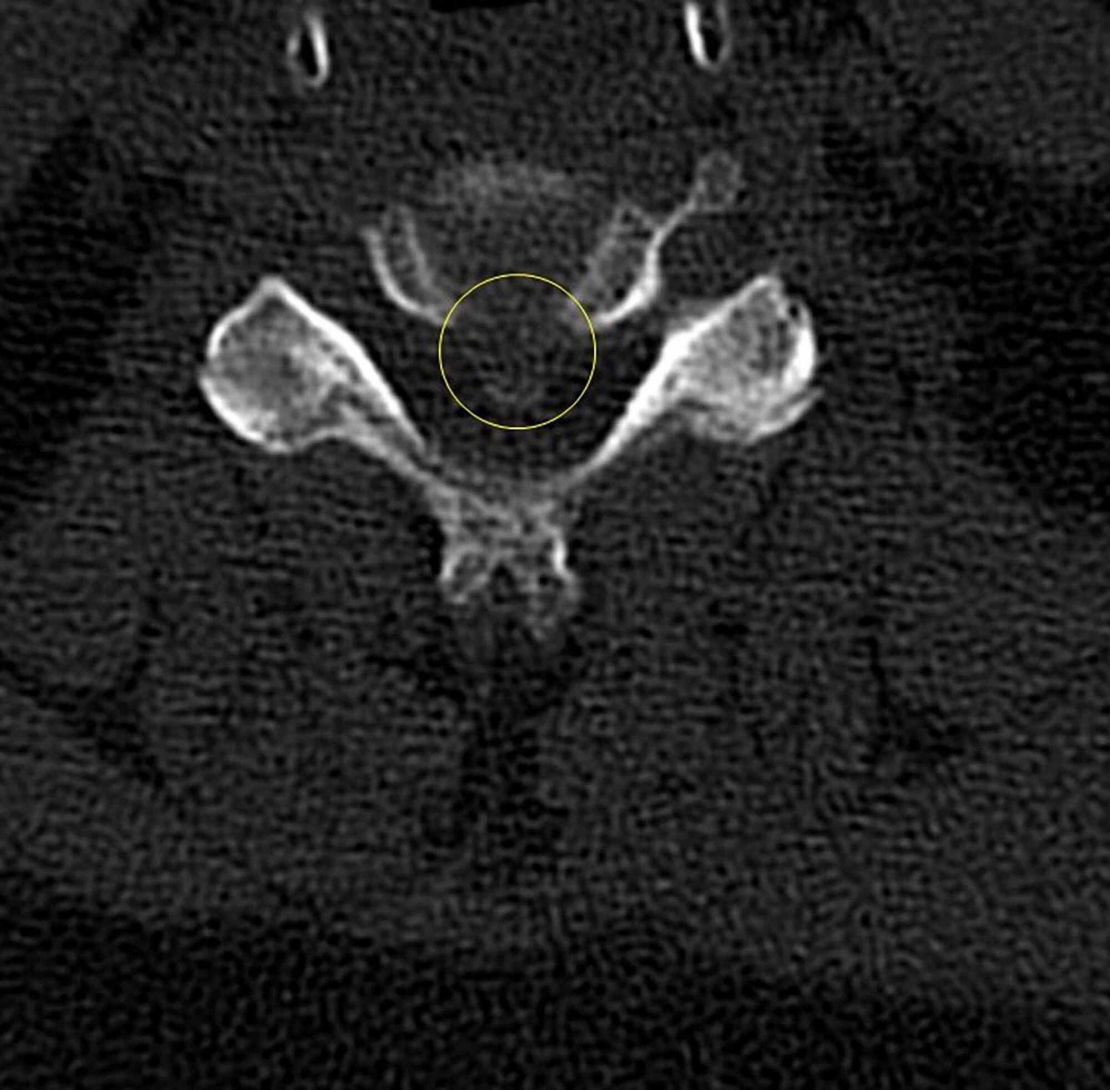
Axial computed tomography scan cut at the level of C5 showcasing severe ossification of the posterior longitudinal ligament (yellow circle) with significant protrusion into the spinal canal.

**Figure 3:**
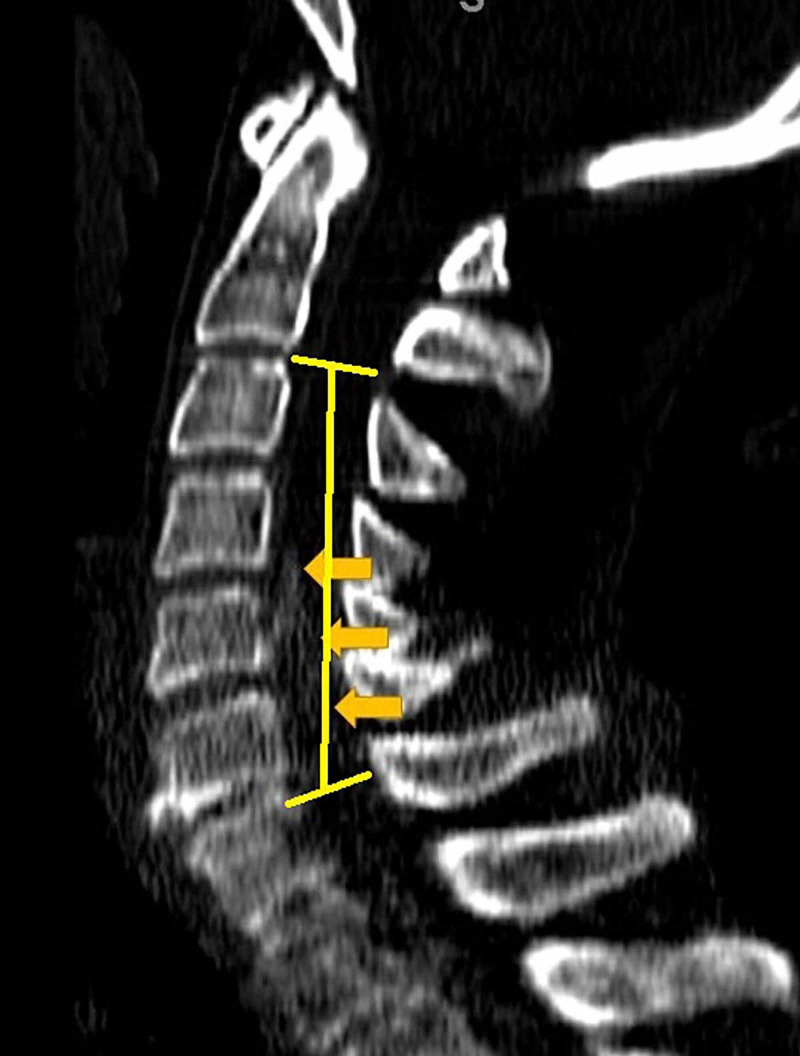
Sagittal computed tomography scan cut of the cervical spine showcasing sever ossification of the posterior longitudinal ligament (orange lines) contacting the K line drawn from C2 to C7.

**Figure 4:**
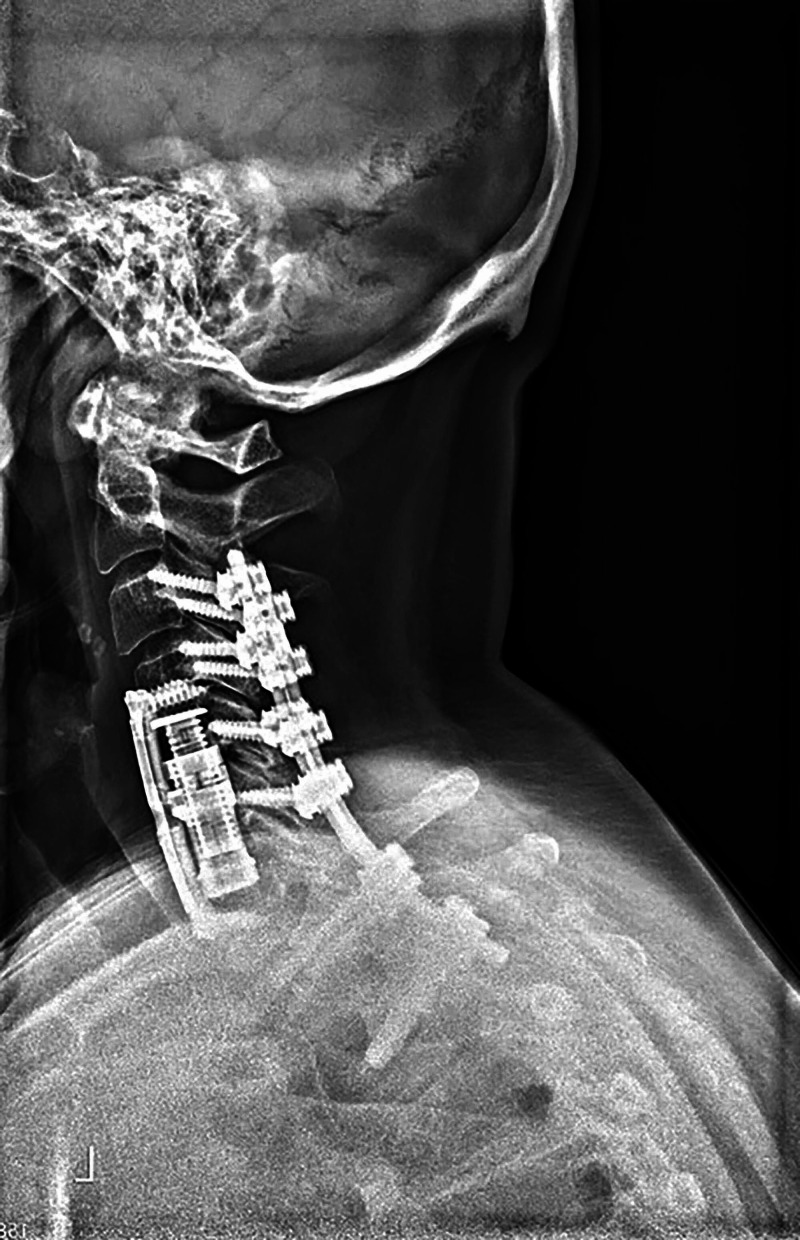
Postoperative lateral standing x-ray showing the anterior and posterior stabilization construct.

A 59-year-old woman was referred to our clinic with complaints of bilateral upper extremity apraxia with ataxic gait for the past three years. She had been clinically diagnosed with MS 20 years prior, and her diagnosis was supported by repeated brain imaging. Her symptoms were initially attributed to her demyelinating disease, but her ataxia had worsened leading to repeated falls during the past year. She had a magnetic resonance imaging scan that showed severe ossification of the posterior longitudinal ligament with spinal cord compression spanning from C4 to C6 that was available 14 months prior to her referral to our spine clinic (Figure 1). A CT scan of her cervical spine showed severe ossification of the posterior longitudinal ligament with calcification posterior to C4, C5 and C6. Figure 2 showcases an axial cut at the level of C5 that illustrates the severe protrusion of the calcification into the canal (yellow circle). Figure 3 showcases an axial cut where the anterior calcification extends beyond the K line spanning from C2 to C7. For these reasons, the decision was made to treat the spinal compression circumferentially to ensure adequate anterior decompression. A corpectomy spanning from C5 to C7 was performed anteriorly, and was reinforced posteriorly with a posterolateral fusion and decompression from C2 to T2 (Figure 4).

Her symptoms improved dramatically after surgery, with resolution of her gait instability, and she cleared physical therapy before her three-month follow-up visit.

## Discussion

In this study, we examined the timeline of management of patients with coexisting demyelinating disorders and cervical spondylosis. We aimed to quantitatively assess the time interval between the initial recognition of myelopathy and radiculopathy symptoms, and the referral to a spine specialist. Additionally, we looked at postoperative complications to ascertain whether patients with DD are at increased risk for complications such as wound breakdown, pseudoarthrosis, and exacerbation of preexisting neurologic deficits. 

Our findings suggest that there is a significant delay in the diagnosis and treatment of symptomatic CS in patients with coexistent DD. The bulk of the delay appears to exist between the initial diagnosis of myelopathy and radiculopathy and the referral to a spine specialist with an average of 67 (m=40) months. Interestingly, the availability of spine imaging showcasing impingement of the neural elements that would warrant a surgical decompression at the time of diagnosis still resulted in a delay in referral by an average of 16.5 months. This likely reflects the difficulty of recognizing spine-specific symptoms in patients who have been experiencing neurological decline for years. While most cases referred to the Spine clinic appear to have undergone surgery early (median of two months), some patients waited months before reconciling with a surgical solution. That is understandable given the fact that physicians are less likely to make a strong argument for surgery if patients have been chronically debilitated, and since their deficit may be partially due to their demyelinating disorder.

Despite the delay in diagnosis and treatment, the vast majority of the patients in our cohort reported positive outcomes with minimal morbidity following surgery. There did not appear to be added risk or complications resulting from surgery compared to the general population. These findings are consistent with recent studies investigating surgical outcomes of patients with concurrent DD, particularly multiple sclerosis, and CS [9]. Our data adds to this body of literature by showcasing that the benefit from surgery persists despite long delays. While nonsurgical management with external cervical immobilization has been recommended in the past for managing patients with coexistent MS and CS [9], more recent analyses have advocated for the safety and effectiveness of surgical decompression [6,7,10,11]. This paradigm shift likely reflects recent advances in the medical management of demyelinating disorders which minimizes postoperative flares of the disease, and also highlights the tremendous improvement in safety and efficacy of spinal instrumentation over the past 20 years. The delays between the diagnosis of myelopathic symptoms and the referral to a spine surgeon may highlight the need to update medical specialists with the latest data on surgical outcomes for this specific patient population.

The timing of diagnosis and treatment of CS also carries a prognostic importance. As suggested by Tetreault et al. in a systematic review of postoperative outcomes after decompression for cervical spondylotic myelopathy, increased severity and duration of symptoms prior to surgery is associated with worse outcomes [12]. These findings are bound to apply in patients with DD. Additionally, a survey-based study by Pope et al. revealed that there was a significant delay in the diagnosis of degenerative cervical myelopathy in the general population, with more than 50% of patients experiencing a delay of more than a year and about 20% experiencing a delay of more than five years from the onset of their symptoms [13]. The data from our current population shows an average diagnostic delay of 99 months (M=52) or 8.25 years, suggesting that DD is a potential risk factor for neglecting spine-related symptoms longer.

While we were able to highlight the benefit of surgical treatment in patients with coexistent DD and CS, our study has some limitations. First, because of the retrospective nature of data review, it is at risk for recall and selection bias. In some patients only the year when the symptoms first started was available, which decreased the accuracy of our estimates. Second, identifying potential spine-related symptoms in the context of this study population was not always clear, which could have resulted in some inaccuracies with estimation of the duration of symptoms. Third, our sample is small and derived from a single institution, which may impact the generalizability of our results. Finally, the pre- and post-operative assessments of patients was partially qualitative except for the neurological exam, and was dependent on patient self-evaluations instead of objective scales [14,15]. The argument could be made that patient-reported outcomes and improvement are paramount, but future studies should prospectively include objective measures of myelopathy.

## Conclusions

There is a significant delay in the diagnosis and treatment of degenerative cervical spondylosis in patients with demyelinating disorders due to overlapping symptoms between the two entities. Despite the delay, surgery remains safe and yields a clinically notable improvement. This calls for increased vigilance amongst medical specialists treating patients with demyelinating disease, and for a lower threshold for ordering cervical spine imaging when symptoms of myelopathy or radiculopathy are present. 
